# Optic nerve sheath meningioma: a case report

**DOI:** 10.1186/1757-1626-1-423

**Published:** 2008-12-29

**Authors:** Savas P Deftereos, Georgios K Karagiannakis, Athina Spanoudaki, Soultana N Foutzitzi, Panos Prassopoulos

**Affiliations:** 1 Department of Radiology, University Hospital of Alexandroupolis, Democritus University of Thrace, Alexandroupolis (Dragana), 68100, Greece

## Abstract

A case of a 75-year old male with right-sided exopthalmos is presented. Outside proptosis of the right eye was initially observed 6 years ago. Opthalmological and endocrinological clinical examination as well as laboratory tests revealed no pathology from optic nerve disc, optic bulb and thyroid related hormones. MRI study demonstrated an optic nerve meningioma. The key imaging findings and the differential diagnosis were discussed in this present paper.

## Case presentation

**Figure 1:**
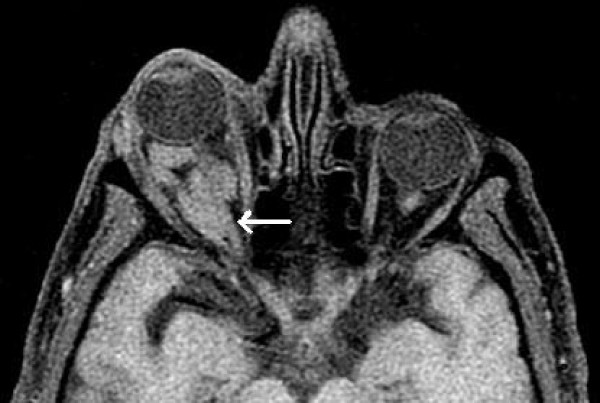
**Axial MR image (T1 with fat saturation)**. An isointense to brain and optic nerve (arrow) lesion which produces exopthalmos. The lesion appears as marked widening along the path of the optic nerve but there is no intracranial extension.

**Figure 2:**
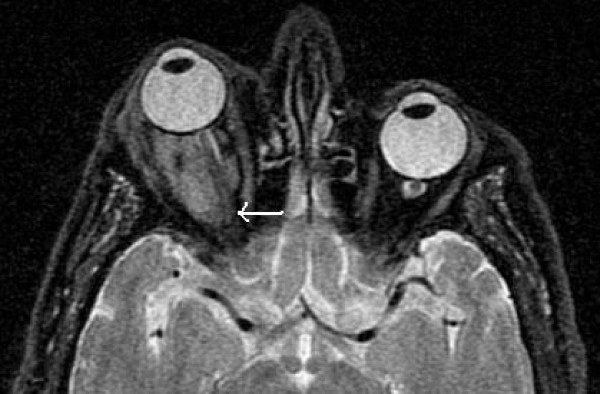
**Axial MR image (T2 with fat saturation)**. The lesion is slightly hyperintense to the optic nerve (arrow).

**Figure 3:**
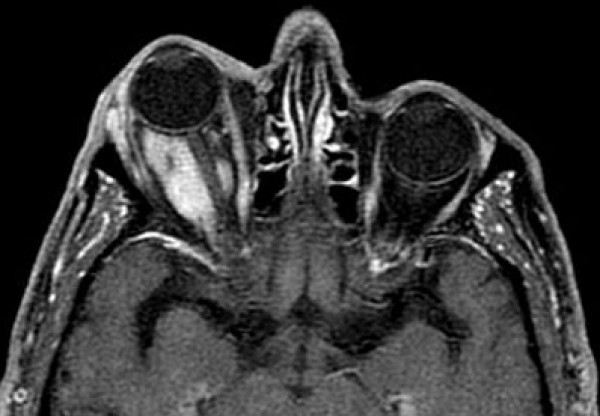
**Axial MR image (T1 with fat saturation) after intravenous administration of paramagnetic media**. There is homogeneous intense enhancement producing a "tram track" appearance around the hypointense optic nerve. Surrounding structures remain intact.

A 75-year old male presents to our department with right-sided exopthalmos. Outside proptosis of the right orbital bulb in comparison with the left was initially observed 6 years ago. The patient visited an ophthalmologist and endocrinologist. Fundoscopic examination revealed no atrophy or oedema of the optic disc and no lesion of the optic bulb. Thyroid related hormones were measured within normal limits. The patient did not proceed to any imaging examinations and returned home. He was under steroid treatment with no response and comes to the hospital nowadays still presenting the same painless symptom (exopthalmos with no prominent change), however claiming visual colour disturbance. Fundoscopy and thyroid hormones still revealed no abnormal findings. MRI study of the orbital cavities was performed. In the MRI protocol T2, T2 with fat saturation, T1, T1 with fat saturation sequences, pre- and post gadolinium injection on axial, sagital and coronal planes were performed. A tubular mass was demonstated in the right orbit showing widening along the length of the nerve sheath and an anterior nerve expansion towards the globe. The mass appeared as isointense to brain and optic nerve tissue on T1 weighted images (Fig. 1) and slightly hyperintense on T2 weighted images (Fig. 2). On T1 weighted images with fat saturation after intravenous administration of paramagnetic substance (gadolinium) the mass presented a homogeneous intense enhancement suggesting in appearance a "tram track" around the hypointense optic nerve (Fig. 3). There is no intracranial extension of the lesion (Fig. 1) or any evidence of surrounding structures invasion (Fig. 3). The presence of optic nerve sheath meningioma was assumed.

## Discussion

Optic nerve sheath meningioma (ONSM) is a term applied to primary and secondary meningioma of the optic nerve. ONSM occurs more commonly in middle aged women. Primary ONSMs account for approximately one third of primary optic nerve tumors and 5% to 10% of orbital tumors [1,2]. Primary ONSM represents a neoplasia of meningothelial cap cells of arachnoid villi and can develop anywhere along the course of the optic nerve. Lesions may be unilateral, bilateral, or multifocal with the latter two subgroups occurring most commonly in patients with type 2 neurofibromatosis [3]. Meningiomas extending from other locations and involving the optic nerve are secondary and may arise from the cavernous sinus, clinoid, sphenoid wing, pituitary fossa, frontal-parietal area or olfactory groove [1].

The diagnosis of OSNM relies heavily on imaging findings. Growth pattern can be either tubular, globular, fusiform or focal. Tubular patterns marked by widening along the length of the nerve sheath are further subdivided into diffuse expansion, apical expansion towards the orbital apex, or anterior expansion towards the globe. Because OSNMs tend to grow outward from the nerve sheath, they are less likely to efface the CSF layer between the nerve and sheath a property that sometimes helps to differentiate meningioma from glioma. Calcification is also a common characteristic of meningioma. MRI currently remains the modality of choice for diagnosis of OSNM, although less sensitive than CT in the recognition of calcification. ONSMs are typically isointense or slightly hypointense to brain and optic nerve tissue on T1 weighted images and hyperintense (may also be hypointense) on T2 weighted images. They present a homogeneous intense enhancement often suggesting in appearane a "tram track" around the hypointense optic nerve in axial sequences. Intracranial extension is rare and, when present, it is restricted in a short distance along the prechiasmatic optic nerve sheath [4,5].

Clinical manifestations include ipsilateral visual loss, color vision disturbance, visual field defect, proptosis, optic disc oedema and motility disturbance.

Main lesions included in the differential diagnosis are optic glioma, orbital pseudotumor, lymphoma. Optic gliomas most commonly occur in children up to 10 years of age. When occurring in adults they present an agressive malignant behaviour (glioblastomas). They are typically either nonenhancing or weakly enhancing tumors, sometimes presenting cystic degeneration. Intracranial extension along the optic nerve pathway is usual. Calcification is rare [6,7].

Orbital pseudotumor is an idiopathic inflammatory process that may involve all intraorbital structures. It shows good clinical response to steroid therapy [6,8].

Primary lymphomas of the orbit are rare most commonly of the non-Hodgkin type [6].

## Conclusion

Although MRI is less sensitive than CT in the recognition of calcification, it currently remains the procedure of choice for diagnosis of ONSM. The imaging findings (MRI) in optic nerve sheath meningioma are quite typical. The benign non invading growth pattern, the quite stable clinical manifestation and the lack of response to steroid therapy strongly suggest the possibility of meningioma. Consequently an aggressive procedure such as biopsy was considered unnecessary when patient's age is relatively advanced and the risk of potential dammage to the optic nerve is high.

## Consent

The authors confirm that informed written consent was received from the patient for publication of the manuscript and figures.

## Competing interests

The authors declare that they have no competing interests.

## Authors' contributions

SD made substantial contributions to analysis and interpretation of data and was a major contributor in writing the manuscript. GK made substantial contributions to conception and design and was a major contributor in writing the manuscript. AS made substantial contributions to acquisition, analysis and interpretation of data. SF made substantial contributions to acquisition, analysis and interpretation of data. PP revised the manuscript critically for important intellectual content. All authors read and approved the final manuscript.
